# Clinical Factors Associated with Disease Severity in Symptomatic Dermographism: A Cross-Sectional Study

**DOI:** 10.3390/medicina62050862

**Published:** 2026-04-30

**Authors:** Özlem Akın, Mehmet Oktay Taşkapan, Asuman Cömert Erkılınç, Özlem Tanrıöver

**Affiliations:** 1 Department of Dermatology, Yeditepe University Faculty of Medicine, Istanbul 34752, Türkiye; oktaytaskapan@hotmail.com (M.O.T.); asuderma@gmail.com (A.C.E.); 2 Department of Medical Education, Marmara University Faculty of Medicine, Istanbul 34752, Türkiye; drozlemtan56@gmail.com

**Keywords:** symptomatic dermographism, physical urticaria, disease severity, chronic inducible urticaria, angioedema

## Abstract

*Background and Objectives*: Symptomatic dermographism (SD) is the most common type of physical urticaria; however, data on its clinical characteristics and factors associated with disease severity remain limited. This study aimed to evaluate clinical features, triggering factors, associated conditions, and factors associated with disease severity in patients with SD. *Materials and Methods*: This cross-sectional study included 174 patients followed at a tertiary dermatology center. Data were collected using a structured questionnaire. Disease severity was categorized as severe versus non-severe using a non-validated, patient-reported classification. Factors associated with severe disease were analyzed using univariate and multivariable logistic regression models. *Results*: The median age was 33.0 years (IQR 27.0–40.2), and 57.4% of patients were female. Severe disease was present in 36.8% of cases. In multivariable analysis, male sex was independently associated with lower odds of severe disease (adjusted OR 0.40, 95% CI 0.20–0.82; *p* = 0.01). Angioedema and psychological stress were associated with disease severity in univariate analyses but not after adjustment. Persistent symptoms were more frequently reported among patients with severe disease. *Conclusions*: Male sex was associated with lower disease severity in this cohort. However, given the cross-sectional design and the use of a non-validated severity assessment, these findings should be interpreted as exploratory and hypothesis-generating, and do not support clinical risk stratification or causal inference.

## 1. Introduction

Symptomatic dermographism (SD) is the most common type of physical urticaria. Recent data on SD revealed a sex- and age-adjusted point prevalence of 3.20% and a lifetime prevalence of 5.94%. The prevalence was higher among females and in the working-age population (25–60 years) [1]. SD is the most common type of chronic inducible urticaria also in children [2]. The data on disease duration and resolution in SD remain limited [3]. Although the duration of SD shows significant variations, the literature indicates that the mean duration of the disease is 6.5 years [4].

SD is characterized by itchy wheals induced by mild stroking, rubbing, or scratching of the skin in the exposed area. Diagnosis is based on a detailed medical history and confirmed by skin provocation testing. Provocation tests include moderate stroking of the volar forearm or upper back skin with a blunt smooth object, dermographic tester, or Fric test. The test response is evaluated 10 min after provocation [5].

Several factors have been reported to be associated with SD, including psychological factors, infections, and medications. Even if there is usually a gradual improvement over several years, the overall course is unpredictable [4,6].

Treatment of the disease is symptomatic, and symptom avoidance is crucial. First-line treatment is the use of second-generation H_1_-antihistamines. If necessary, the dose can be increased up to fourfold. Omalizumab is the most effective medication in cases of resistance. Narrow-band UVB, H_2_-antihistamines, and cyclosporin A are other options, although evidence from controlled trials is missing [3].

Although SD is the most common form of physical urticaria, data regarding clinical characteristics and factors associated with disease severity remain limited. Therefore, this study aimed to evaluate the clinical characteristics, triggering factors, associated conditions, and patient-reported disease course in patients with SD and to identify factors associated with disease severity.

## 2. Materials and Methods

This was a cross-sectional descriptive study with analytical components, conducted in patients diagnosed with SD. Patients who had been followed at our center since 2008 were consecutively identified from a database maintained by the Department of Dermatology and Venereology at Yeditepe University School of Medicine. Data were collected at a single time point using a structured questionnaire.

The questionnaire was developed by the authors based on clinical experience and relevant literature. Although not formally validated, it was structured to ensure consistency in data collection. It included items on demographic characteristics, clinical features, disease course, treatment modalities, coexisting urticaria types, atopic/allergic diseases, angioedema, associated systemic conditions (e.g., hepatitis, thyroid disease, rheumatological diseases), menopausal status, and potential triggering factors such as infections, medications, systemic diseases, and stressful life events.

This study was approved by the Institutional Ethics Committee of Marmara University (approval number: 09.2026.26-0061; approval date: 20 January 2026) and conducted in accordance with the Declaration of Helsinki. Written informed consent was obtained from all participants.

A total of 240 patients diagnosed with SD by provocation testing (stroking the back skin with a tongue blade) were initially identified. Of these, 181 patients were successfully contacted, and 174 agreed to participate (response rate: 72.5%; 174/240). All participating patients completed the questionnaire.

Age and disease duration were recorded as continuous variables. Disease duration was calculated in months by combining reported years and months.

Since there is no standardized scoring system in SD, and objective assessment tools such as a dermographometer or FricTest were not available in our setting, disease severity was assessed based on patient-reported symptom intensity during untreated periods.

Severity categories were defined as follows: mild (occasional symptoms with minimal discomfort not interfering with daily activities), moderate (frequent symptoms with noticeable discomfort partially affecting daily activities), and severe (persistent or intense symptoms significantly impairing daily activities).

This classification was based on routine clinical evaluation in daily practice. Although a validated scoring system was not used, this approach reflects routine clinical assessment; however, it may limit reproducibility and introduce subjectivity. This approach may also affect internal validity and should be interpreted with caution. Formal internal consistency or sensitivity analyses were not performed, given the retrospective design and the use of a non-validated, patient-reported classification.

For regression analyses, severity was dichotomized as severe vs. non-severe (mild/moderate).

Binary variables included sex, presence of angioedema, atopy, family history of urticaria, persistence of symptoms, and reported triggering factors such as work-related stress or major examinations.

Continuous variables were summarized as mean ± standard deviation or median (interquartile range, IQR), depending on distribution. Categorical variables were presented as numbers and percentages.

Comparisons between patients with severe and non-severe disease were performed using the Mann–Whitney U test for continuous variables and chi-square or Fisher’s exact test for categorical variables, as appropriate.

A multivariable logistic regression model was constructed to identify factors associated with severe disease. Variables were selected based on clinical relevance and results of univariate analyses, while maintaining an appropriate events-per-variable ratio to minimize the risk of overfitting. The events-per-variable ratio was approximately 10, which is generally considered acceptable to limit overfitting.

Multicollinearity among independent variables was assessed using variance inflation factors (VIF), with values < 5 considered acceptable. Model fit was evaluated using the −2 log likelihood, Nagelkerke R^2^, and the Hosmer–Lemeshow goodness-of-fit test. A non-significant Hosmer–Lemeshow test was interpreted as indicating good model fit.

Effect sizes were reported as odds ratios (OR) with 95% confidence intervals (CI). Statistical significance was defined as a two-sided *p*-value < 0.05. Analyses were performed using SPSS version 25.0 (IBM Corp., Armonk, NY, USA).

Cases with missing key variables were excluded from the analysis. The proportion of missing data was low and unlikely to affect the results.

This study was conducted and reported in accordance with the Strengthening the Reporting of Observational Studies in Epidemiology (STROBE) guidelines for observational studies.

## 3. Results

### 3.1. Patient Characteristics

Of the 240 patients screened, 174 with SD (100 females, 74 males) participated in the study. The median age was 33.0 years (IQR 27.0–40.2; range 10–69 years). The demographic and clinical characteristics of patients are shown in Table 1. At the time of questionnaire completion, 108 patients (62.1%) were still symptomatic. Overall, 103 patients (59.2%) reported at least one disease-free period during the course of SD. Disease severity was classified as mild in 50 (22 female) patients (28.7%), moderate in 60 (32 female) patients (34.5%), and severe in 64 (46 female) patients (36.8%).

### 3.2. Comparison of Clinical Characteristics by Disease Severity

Severe disease was more common in female than in male patients (46 [71.9%] vs. 18 [28.1%], *p* = 0.03). A higher proportion of patients with severe disease reported ongoing symptoms at the time of assessment, although disease duration did not differ significantly.

Of the 108 symptomatic patients, 66 (61.1%) were receiving treatment at the time of evaluation. Second-generation H1-antihistamines were used by 62 patients (93.9%), and 58 (93.5%) reported marked clinical improvement. Possible or suspected triggering factors were reported by 107 patients (61.5%). The most frequently reported triggering factor was stressful life events (n = 84), followed by medications (n = 11, 6.3%). Detailed information on triggering factors is presented in Table 2.

Medications potentially associated with symptom onset, disease duration, and severity are summarized in Supplementary Table S1.

Infectious or inflammatory comorbid conditions were identified in 54 patients (31.0%), including thyroid diseases, sinusitis, hepatitis, and rheumatological disorders. Other inflammatory conditions, such as gastritis, pericarditis, uveitis, and chronic cystitis, were reported in 12 patients (6.9%). Fourteen of 100 female patients were in the menopausal period; one reported a temporal association between menopause and SD onset. Concomitant atopic/allergic diseases diagnosed by a physician were present in 37.9% (66/174) of patients.

Seventeen patients (9.8%) had another type of urticaria in addition to SD, including cold-induced urticaria (n = 8), pressure urticaria (n = 4), and chronic spontaneous urticaria (n = 6). One patient had concomitant SD, chronic spontaneous urticaria, and cold urticaria.

Angioedema during the symptomatic period was reported by 19 patients (10.9%), including 17 females and 2 males. Angioedema was more frequently reported in symptomatic female patients than in symptomatic male patients (*p* = 0.003). Univariate comparisons between patients with severe and non-severe SD are shown in Table 3. In univariate analysis, the presence of angioedema was associated with severe disease (OR 3.40, 95% CI, 1.26–9.14; *p* = 0.01).

Other variables, including atopy, work-related stress, family history, and symptom duration, were not significantly associated with disease severity. Multivariable logistic regression analysis results are presented in Table 4. The multivariable logistic regression model demonstrated an acceptable fit to the data (−2 log likelihood = 228.915). The explanatory power of the model was modest (Nagelkerke R^2^ = 0.101). The Hosmer–Lemeshow goodness-of-fit test was non-significant (χ^2^ = 7.43, *p* = 0.267), indicating good model fit. Multicollinearity diagnostics showed no evidence of collinearity among independent variables (maximum VIF = 1.11). Male sex remained independently associated with lower odds of severe disease in this cohort (adjusted OR 0.40, 95% CI, 0.20–0.82; *p* = 0.01). No other variables included in the model showed a statistically significant association with severe disease.

## 4. Discussion

SD has been recognized for nearly a century, but its cause remains largely unknown. Reports in the literature indicate that, in some patients, injecting their serum into healthy volunteers induces wheals after scratching. Specific IgE to autoallergens generated by exposing the skin to shear force could play a role in the pathomechanism in some SD patients [3]. Over the past five years, several studies have provided new insights into the etiopathogenesis of SD, including evidence of central nervous system involvement, alterations in gut microbiota, and the identification of novel clinical variants [7,8,9,10,11,12].

One of the findings of this study was that male sex was associated with lower odds of severe disease; however, this observation should be interpreted with caution. Given the cross-sectional design, causal relationships cannot be established, and this association may be influenced by residual confounding factors.

The observed association may also reflect differences in symptom perception or reporting, healthcare-seeking behavior, or characteristics of the study population. In addition, the relatively limited sample size may have restricted the ability to fully adjust for potential confounders.

Although sex-related differences in mast cell regulation and hormonal influences have been suggested in previous studies [13,14,15], these mechanisms were not assessed in the present study and therefore remain speculative.

Other variables, including age, atopy, work-related stress, and angioedema, were not independently associated with disease severity after multivariable adjustment.

Given the cross-sectional design, both exposure variables and disease severity were assessed simultaneously, precluding any temporal or causal inference. The observed associations may be influenced by residual confounding, selection bias, and potential reverse causation.

Angioedema resulting from mechanical stimulation in SD remains a controversial issue. In the literature, some reports indicate that SD is associated with angioedema in 10–21% of cases [4,16]. In our study, 19 patients (10.9%) reported angioedema during the symptomatic period. Although angioedema appeared to be more frequent among female patients (89.5%), this observation should be interpreted with caution given the limited number of cases.

While angioedema was significantly associated with severe disease in univariate analysis, this association did not persist in multivariable analysis. These findings suggest that angioedema may be associated with disease severity; however, it does not appear to be an independent factor in this cohort. Given the cross-sectional design, these observations should be interpreted cautiously and considered hypothesis-generating.

Although psychological factors have been implicated in SD, it remains one of the most common forms of physical urticaria reported to be exacerbated by life events and emotional stress [17]. Taskapan et al. reported a close temporal association between SD and psychological factors [6], while Wallengren et al. showed that acute psychosocial stress did not significantly alter dermographic responses, although a substantial proportion of patients reported impaired quality of life and identified stress as a precipitating factor [18]. Similarly, in a questionnaire-based study, stress-related episodes were frequently reported among patients with SD [4].

In our cohort, 48.3% of patients reported symptom onset following a stressful life event, whereas others did not identify stress as a trigger. However, no statistically significant differences were observed between these groups in terms of disease duration or severity.

These findings suggest that psychological stress may be a commonly reported triggering factor; however, its relationship with disease severity or persistence remains unclear. Given the reliance on patient-reported data, recall bias cannot be excluded, and the cross-sectional design precludes causal inference.

In this study, the mean age of the 174 patients with SD at diagnosis was 35 years, and the mean disease duration was 4.3 years. Although this duration is somewhat shorter than that reported in the literature [17,19,20], variability in disease course across studies should be considered.

At the time of assessment, 108 patients remained symptomatic, with a mean reported disease duration of approximately 6.2 years. These findings may reflect the persistence of symptoms in a substantial proportion of patients; however, given the cross-sectional design, no conclusions can be drawn regarding long-term disease course or prognosis.

Patient-reported disease course indicated that a substantial proportion of patients continued to experience symptoms at the time of assessment, particularly those with severe disease. Disease duration and symptom persistence were based on patient self-report at a single time point and should not be interpreted as objective prognostic outcomes. Longitudinal studies are needed to clarify the natural history and long-term outcomes of SD.

Data on the relationship between SD and medications are limited. SD has been reported in association with systemic penicillins, famotidine, atorvastatin, and cephalosporins, as well as progesterone and topical diphenylcyclopropenone [6,21,22].

In our study, 11 patients reported symptom onset following medication use (Supplementary Table S1). Most patients described an acute onset of urticarial symptoms temporally related to drug exposure, followed by persistent skin reactivity compatible with SD.

Although some of these patients had severe disease, the small number of cases precludes any meaningful interpretation regarding disease severity or duration. Therefore, these observations should be interpreted with caution and considered descriptive and hypothesis-generating.

No causal relationship between medication use and SD severity or duration can be established based on our data. Further large-scale, prospective studies are needed to better clarify the potential role of medications in the onset and clinical course of SD.

Although the etiopathogenetic role of atopy remains unclear, some reports indicate that certain types of chronic inducible urticarias (especially cholinergic urticaria and cold urticaria) may be associated with atopic/allergic diseases [23]. The literature on the role of atopy in SD is limited, and the relationship between the two remains uncertain. However, a few studies have reported a relatively high prevalence of atopy and/or atopic diseases, including aeroallergen sensitization, in patients with SD (38–61.7%) [24,25,26,27].

In our study, 66 patients (37.9%) had atopic/allergic diseases. No statistically significant association was observed between atopy and either disease severity or duration. Although a history of atopy was more frequent among patients with severe disease in univariate analyses, it did not retain independent significance in the multivariable model.

These findings indicate that atopy may coexist with SD; however, no independent association with disease severity was demonstrated in this cohort. Given the cross-sectional design, these observations should be interpreted cautiously.

The role of infections as potential triggering factors in SD remains uncertain. To date, no study has clearly established a causal relationship between infections and the development of SD. In our cohort, a small number of patients reported a temporal association between the onset of SD and concurrent infections, including infectious mononucleosis, toxoplasmosis, vaginal candidiasis, and upper respiratory tract infection.

In contrast, a range of other infectious and inflammatory conditions (e.g., thyroid disorders, sinusitis, hepatitis, gastritis, pericarditis, uveitis, and rheumatological diseases) were also observed in a subset of patients but were not temporally associated with SD onset.

Previous reports have suggested that infections such as infectious mononucleosis and toxoplasmosis may be associated with acute or inducible forms of urticaria [28,29,30]; however, their specific role in SD remains unclear.

Given the limited number of cases and the reliance on patient-reported temporal associations, these observations should be interpreted with caution. No causal relationship can be established, and the findings should be considered exploratory. Further studies are needed to better clarify the potential role of infections in the pathogenesis of SD.

In our cohort, a small number of patients reported the onset of SD following various inflammatory or allergic reactions, including food allergy, bee stings, insect bites, sunburn, and hair dye reactions. These patients described a temporal relationship between the triggering event and the onset of symptoms.

In several cases, patients reported an initial episode resembling acute urticaria, followed by persistent skin reactivity compatible with SD. However, given the limited number of cases and the reliance on patient-reported data, these observations should be interpreted with caution.

No causal relationship can be established, and these findings should be considered exploratory. Further studies are needed to clarify whether such inflammatory triggers may play a role in the initiation of SD.

Two or more types of chronic urticaria, including inducible forms, may coexist [4]. Similarly, in this study, 17 patients had another type of urticaria associated with SD. However, these associations did not seem to affect the severity, duration, and prognosis of SD.

This study has several limitations. First, its cross-sectional design precludes any causal or temporal inferences, and the observed associations should be interpreted accordingly. Second, disease severity was assessed using a non-validated, patient-reported classification, which may introduce subjectivity, limit reproducibility, and affect internal validity. The Urticaria Activity Score (UAS), a validated tool commonly used in chronic spontaneous urticaria, is not directly applicable to symptomatic dermographism, for which no standardized or validated severity assessment instrument currently exists [3]. In addition, the absence of internal consistency or sensitivity analyses may further limit the robustness of the severity classification. Additionally, the use of patient-reported severity may have influenced subgroup comparisons and regression estimates.

Third, several variables, including triggering factors, stress, and disease duration, were based on retrospective self-report and are therefore subject to recall bias. In addition, clinical characteristics at the time of diagnosis and those reported at the time of the survey may not fully correspond, which could have influenced the accuracy of patient-reported outcomes.

The study was conducted in a single tertiary care center, which may have led to selection bias and an overrepresentation of patients with more severe or persistent disease, thereby limiting generalizability. Furthermore, only 174 out of 240 eligible patients participated, raising the possibility of non-response bias. A comparison between responders and non-responders was not possible due to limited available data.

Finally, the relatively small number of cases in certain subgroups (e.g., medication- or infection-related onset) limits the strength of subgroup analyses, and the lack of objective assessment tools for dermographism severity represents an additional limitation. Model diagnostics were performed; however, given the relatively limited number of outcome events, these results should be interpreted with caution.

## 5. Conclusions

The evaluation of data obtained from this study indicated that sex and several clinical features, including angioedema and psychological stress, were associated with disease severity; however, only male sex remained independently associated with lower disease severity after multivariable adjustment.

Given the cross-sectional design, these findings should be interpreted as associations observed in this cohort rather than causal relationships. The results highlight potential clinical correlates of disease severity, but further prospective studies are needed to confirm these observations and clarify their clinical implications.

## Figures and Tables

**Table 1:** Demographic and clinical characteristics of patients with symptomatic dermographism (SD).

Variables	Total (*n* = 174)	Female (*n* = 100)	Male (*n* = 74)
Age (years), median (IQR)	33.0 (27.0–40.2)	33 (27.0–42.0)	34 (28.0–40.0)
Currently symptomatic, *n* (%)	108 (62.1%)	59 (59%)	49 (66.2%)
Symptom–free at survey, *n* (%)	66 (37.9%)	41 (41%)	25 (33.8%)
Disease duration (years), mean ± SD	4.3 ± 5.2	4.4 ± 5.7	4.3 ± 4.4
Disease duration in symptomatic patients (years), mean ± SD	6.2 ± 5.4	6.7 ± 6.1	5.5 ± 4.3
Disease duration in symptom-free patients (years), mean ± SD	1.28 ± 2.83	0.98 ± 2.58	1.76 ± 3.18
Angioedema, *n* (%)	19 (10.9%)	17 (17.0%)	2 (2.7%)
Concomitant atopic/allergic disease *, *n* (%)	66 (37.9%)	33 (33.0%)	33 (44.6%)
Other types of urticaria, *n* (%)	17 (9.8%)	15 (15.0%)	2 (2.7%)

* Asthma, allergic rhinitis, atopic dermatitis (physician-diagnosed). Values are presented as n (%) for categorical variables and mean ± SD or median (IQR) for continuous variables, as appropriate.

**Table 2:** Possible and/or suspected triggering factors in SD patients (*n* = 174).

Triggering Factor	*n*, %
Any triggering factor reported	107 (61.5)
Stressful life events	84 (48.2)
Multiple stressful events	10 (5.7)
Medications	11 (6.3)
Infections	4 (2.2)
Food-induced urticaria	3 (1.7)
Bee sting	2 (1.1)
Insect bite	1 (0.5)
Severe sunburn	1 (0.5)
Hair dye	1 (0.5)

**Table 3:** Clinical characteristics according to disease severity (univariate analysis).

Variable	Severe/Total (Yes)	Severe/Total (No)	OR (95% CI)	*p*
Atopy (yes)	29/64 (45.3%)	35/110 (31.8%)	1.63 (0.87–3.07)	0.12 *
Angioedema (yes)	12/19 (63.2%)	52/155 (33.5%)	3.40 (1.26–9.14)	0.01 *
Work–related stress/important exam (Yes)	15/35 (42.9%)	49/139 (35.3%)	1.38 (0.65–2.93)	0.40 *
Family history (Yes)	16/38 (42.1%)	48/136 (35.3%)	1.33 (0.64–2.78)	0.44 *
Symptom duration (months), median (IQR)	5.0 (1.0–11.0)	3.0 (0.8–24.0)	—	0.47 **
Sex (male)	18/64 (28.1%)	56/110 (50.9%)	0.37 (0.19–0.73)	0.003 *
Age (years), median (IQR)	30.0 (26.3–38.8)	34.5 (28.0–43.3)	—	0.057 **

Values are presented as *n* (%) for categorical variables and median (IQR) for continuous variables, as appropriate. * χ^2^ test, ** Mann–Whitney U test.

**Table 4:** Multivariable logistic regression analysis of factors associated with severe disease.

Variables	OR (95% CI)	*p*
Atopy (yes)	1.77 (0.89–3.53)	0.10
Angioedema (yes)	2.20 (0.77–6.29)	0.13
Work stress (yes)	1.35 (0.59–3.04)	0.47
Family history (yes)	1.17 (0.52–2.64)	0.69
Male sex	0.40 (0.20–0.82)	0.01
Age (year, continuous)	0.98 (0.95–1.01)	0.14

## Data Availability

The datasets generated and/or analyzed during the current study are available from the corresponding author on reasonable request.

## Supplementary Materials

The following supporting information can be downloaded at: https://www.mdpi.com/article/10.3390/medicina62050862/s1, Table S1. Clinical characteristics of patients with medication-related symptomatic dermographism.

## Abbreviations

The following abbreviations are used in this manuscript:

SD	Symptomatic dermographism
OR	Odds ratio
CI	Confidence interval
IQR	Interquartile range
