# Supplementary material for: Clinical Factors Associated with Disease Severity in Symptomatic Dermographism: A Cross-Sectional Study
Source: Medicina (Kaunas). 2026 Apr 30;62(5):862. doi: 10.3390/medicina62050862 (PMC13208245; doi:10.3390/medicina62050862)
Supplement: Supplementary file 1 [file medicina-62-00862-s001.zip › medicina-4241527-supplementary.pdf]

Supplementary Table S1. Clinical characteristics of patients with medication-related symptomatic dermographism					
**Patient No:**	**Age of SD onset**	**Sex**	**Medication**	**Duration of SD**	**Severity**
1	42	M	Antihypertensive (Not specified)	10 months	Severe
2	23	F	Cephalosporin	30 years	Severe
3	21	F	Fluconazole	1 week	Severe
4	17	M	Cephalosporin	1 week	Moderate
5	68	M	Naproxen sodium	3 days	Moderate
6	25	F	Cephalosporin	1 week	Severe
7	46	F	Paracetamol	1 year	Severe
8	40	M	Vitamin B 12 supplement	6 months	Severe
9	60	F	Paracetamol + propyphenazone + caffeine	2 weeks	Severe
10	29	F	Paracetamol + pseudoephedrine + dextromethorphan	5 months	Moderate
11	56	F	Feniramidol	13 months	Moderate
